# Assessing hydrocarbon degradation capacity of *Isoptericola peretonis* sp. nov. and related species: a comparative study

**DOI:** 10.3389/fmicb.2025.1471121

**Published:** 2025-02-05

**Authors:** Àngela Vidal-Verdú, Adriel Latorre-Pérez, Javier Pascual, Ruth Mañes-Collado, Aitana Nevot-Terraes, Manuel Porcar

**Affiliations:** ^1^Institute for Integrative Systems Biology I2SysBio (Universitat de València-CSIC), Paterna, Spain; ^2^Darwin Bioprospecting Excellence SL. Parc Científic Universitat de València, Paterna, Spain

**Keywords:** *Isoptericola*, new species, hydrocarbon degradation, biosurfactants, bioremediation

## Abstract

Since the beginning of their production and use, fossil fuels have affected ecosystems, causing significant damage to their biodiversity. Bacterial bioremediation can provide solutions to this environmental problem. In this study, the new species *Isoptericola peretonis* sp. nov. 4D.3^T^ has been characterized and compared to other closely related species in terms of hydrocarbon degradation and biosurfactant production by in vitro and in silico analyses. Biosurfactants play an important role in microbial hydrocarbon degradation by emulsifying hydrocarbons and making them accessible to the microbial degradation machinery. The tests performed showed positive results to a greater or lesser degree for all strains. In the synthesis of biosurfactants, all the strains tested showed biosurfactant activity in three complementary assays (CTAB, hemolysis and E_24_%) and rhamnolipid synthesis genes have been predicted in silico in the majority of *Isoptericola* strains. Regarding hydrocarbon degradation, all the *Isoptericola* strains analyzed presented putative genes responsible for the aerobic and anaerobic degradation of aromatic and alkane hydrocarbons. Overall, our results highlight the metabolic diversity and the biochemical robustness of the *Isoptericola* genus which is proposed to be of interest in the field of hydrocarbon bioremediation.

## Introduction

Hydrocarbons, derived primarily from fossil fuels, are one of the major pollutants on Earth and are ubiquitous due to industrial activities, transportation, and accidental spills. Given their hydrophobic nature, these are very recalcitrant compounds that persist into ecosystems and considerably pollute soils, aquifers, and marine environments (Daher Hazaimeh and Ahmed, 2021; Lien et al., 2014; Wang et al., 2021). Therefore, microbial communities from polluted sites are shaped by the prolonged exposure to hydrocarbons. On top of that, not only natural environments are affected, which have been the main focus for studies on hydrocarbon pollution (Acosta-González et al., 2015; Cabral et al., 2021; Jimoh et al., 2022), but also urban artificial environments such as gas stations, fuel tanks and car tank components that have been shown to harbor adapted microbial communities to the presence of fuels (Bücker et al., 2014; da Fonseca et al., 2019; Lima et al., 2019; Vidal-Verdú et al., 2022).

In this context, bioremediation and bioprospecting arise together to address this critical issue. On the one hand, bioremediation, understood as the intervention aimed at alleviating pollution by exploitation of biological activities either by natural attenuation, bio-stimulation or bio-augmentation (de Lorenzo, 2008), is a cost-effective, environmentally friendly and minimally invasive technique when applied in situ compared to traditional ex situ contaminant removal techniques (Orellana et al., 2017; Perelo, 2010). On the other hand, hydrocarbon-degrading microorganisms occur naturally in ecosystems, constituting less than 1% of the total microbial communities under normal conditions. When there is an increase in the presence of petroleum hydrocarbons, they can account for up to 10% of the total microbial communities (Atlas, 1995). Hence, bioprospecting of oil-polluted sites is a directed strategy aimed at discovering hydrocarbon-degrading microorganisms or activities for bioremediation.

In addition, surface-active molecules (biosurfactants) play a key role by emulsifying and thereby enhancing the bioavailability of highly hydrophobic molecules. This process is critical for microbial access and subsequent degradation of hydrocarbons, highlighting the importance of harnessing bacterial biosurfactant production for enhanced bioremediation projects (Elumalai et al., 2021).

Hydrocarbon-polluted environments, especially artificial urban niches, which have not been studied much to date, may constitute a biodiversity hotspot. This proved true in our previous bioprospecting study of the car tank lid in which the environmental strain 4D.3^T^, tentatively identified as *Isoptericola* sp., was found to degrade diesel (Vidal-Verdú et al., 2022). To the extent of our knowledge, before our finding, only the strain *I. chiayiensis* 103-Na4 had been characterized in certain detail as biosurfactant producer and being able to degrade crude oil (Lee et al., 2018a), phenanthrene and pyrene (Lee et al., 2018b). Other studies had also pointed out the ability of other *Isoptericola* strains to play a role in hydrocarbon degradation (Ahmad et al., 2021; Al-Mailem et al., 2015; Polivtseva et al., 2020; Radwan et al., 2010). Along with this fuel catabolic potential, strains from this genus have also been described as possessing industrially relevant features such as cellulolytic (Bae et al., 2021), alginate lyase (Chen et al., 2018), and chitinase (Wu et al., 2011) enzymatic activities as well as antimicrobial (Girão et al., 2019; Kaushik et al., 2021) and plant-growth promoting capabilities (Alghamdi et al., 2023) and polyhydroxybutyrate (PHB) synthesis (Krishnan et al., 2021).

The genus *Isoptericola* was first described by Stackebrandt et al. (2004) who reclassified *Cellulosimicrobium* var*iable* MX5^T^, a cellulolytic and xylanolytic strain that differed substantially from the type species of the genus *Cellulosimicrobium* (Bakalidou et al., 2002), to *I. variabilis.* At the time of writing, this genus is composed of 14 species, 11 with validly published names,^1^ and belongs to the family *Promicromonosporaceae* and the *Actinomycetes* class. The isolation environments of the members of this genus have been very diverse, including the hindgut of a termite (Bakalidou et al., 2002), a Roman catacomb (Groth et al., 2005), the root tissue of a cucumber (Kämpfer et al., 2016) and, mainly, soil samples (Bing et al., 2024; Huang et al., 2012; Kaur N. et al., 2014; Kumar et al., 2021; Tseng et al., 2011; Yoon et al., 2006) and saline sediments (Guan et al., 2013; Ming et al., 2020; OuYang et al., 2023; Wu et al., 2010; Zhang et al., 2005).

Therefore, we now describe here, by a polyphasic approach, a new species of the genus *Isoptericola* isolated from an urban hydrocarbon-polluted environment, the car tank lid, for which the name *Isoptericola peretonis* sp. nov. is proposed. On top of that, we also compare by both experimentally and genomic analysis, the new species and several species from the genus *Isoptericola,* on their ability to produce biosurfactants by in vitro assays as well as to degrade various hydrocarbons following in silico analyses, in order to characterize the bioremediation potential of this poorly known genus. This study reveals that species from the genus *Isoptericola* have outstanding potential as chassis for biotechnological applications, mainly on bioremediation, especially hydrocarbon remediation, since they show high physicochemical robustness and great metabolic diversity.

## Materials and methods

### Strain isolation

Strain 4D.3^T^ was isolated during a previous study of the microbiota on a car tank lid and its ability to degrade diesel (Vidal-Verdú et al., 2022). Briefly, sampling was carried out in the parking areas of the Institute for Integrative Systems Biology (I2SysBio; Paterna, Spain). Strain 4D.3^T^ was isolated after an enrichment process using growth on a basal medium supplemented with diesel as the main carbon source as a selection pressure. The enriched culture consisted of the inoculation of 3 mL of Minimal Medium (Composition in g/L: 2 NaNO_3_, 1 K_2_HPO_4_, 0.5 MgSO_4_·7H_2_O, 0.5 KCl, 0.5 sucrose) supplemented with diesel 10% (v/v) with the dust sampled from a car tank lid. Cultures were incubated at 30°C and 120 rpm for 1 week. Afterwards, 25 μL of the culture were transferred to new fresh media and this process was repeated weekly for 4 weeks. After the enrichment period, several dilutions of the culture were carried out in Phosphate Buffer Saline (PBS, composition in g/L: 8.0 NaCl, 0.2 KCl, 1.44 Na_2_HPO_4_, 0.24 KH_2_PO_4_; adjusted to pH 7.4) and inoculated on Lysogeny Broth Agar (LB, composition in g/L: 10.0 tryptone, 10.0 NaCl, 5.0 yeast extract, 15.0 agar). Plates were incubated for 2 weeks at 30°C and isolated colonies were purified, presumptively identified by 16S rRNA gene sequencing and cryopreserved in 20% glycerol (v/v).

### 16S rRNA gene sequencing

The whole 16S rRNA gene from 4D.3^T^ was amplified by PCR using primers 8F (5′-AGAGTTTGATCCTGGCTCAG-3′) and 1492R (5′-GGTTACCTTGTTACGACTT-3′) and sequenced by Sanger sequencing with these two primers as well as the internal primers 1055F (5′- ATGGCTGTCGTCAGCT-3′) and 341R (5′-CTGCTGCCTCCCGTAGG-3′). The nearly complete 16S rRNA gene sequence (1,356 bp, accession number MZ562363) was analyzed by the EzBioCloud database tool^2^ and the most closely related type strains were acquired from the DSMZ (Leibniz Institut, Deutsche Sammlung von Mikroorganismen und Zellkulturen GmbH, Germany) as reference strains for the comparative study: *Isoptericola cucumis* DSM 101603^T^; *Isoptericola* var*iabilis* DSM 10177^T^; *Isoptericola nanjingensis* DSM 24300^T^; *Krasilnikoviella flava* DSM 21481^T^.

### Genome sequencing, assembly and annotation of *I. peretonis* sp. nov. 4D.3^T^, *I. nanjingensis* and *I. variabilis* strains

Genomic DNA extraction of all strains was carried out from the recovered biomass of an overnight culture of each strain on the suggested culture medium by DSMZ (DSMZ medium 92: Tryptic Soy Agar) (TSA, composition in g/L: 15.0 Tryptone, 5 Soya peptone, 5 NaCl, 15 Agar) at 30°C and DNeasy Power Soil kit (Qiagen) was used by following manufacturer’s instructions. DNA concentration was measured by Qubit x1 dsDNA HS Assay Kit (Qubit 2.0 Fluorometer, Thermo Fisher, Waltham, United States).

Genome sequencing was carried out with the NovaSeq 6000 system (Illumina), and the quality of sequence reads was evaluated with the FastQC tool (v. 0.11.5). SPAdes (v. 3.14.1) was utilized for genome assembly, and QUAST (v. 5.0.2) and CheckM (v. 1.1.3) were employed to calculate assembly statistics and evaluate completeness and contamination levels, respectively. Genome annotation was performed both with prokka (Seemann, 2014) and the RAST tool kit (RAStk) integrated in PATRIC v. 3.6.8.^3^ TYGS (Meier-Kolthoff and Göker, 2019) was used to identify the most closely related type strains to 4D.3^T^ with publicly available genomes and to calculate the digital DNA–DNA hybridization (dDDH) index, while ANI was computed using FastANI (v. 1.33) (Jain et al., 2018).

### Phylogenetic and phylogenomic analysis

Based on the 16S rRNA gene sequences, the phylogenetic reconstruction was obtained by maximum-likelihood (ML) (Felsenstein, 1981) and neighbor-joining (NJ) (Saitou and Nei, 1987) methods with the software MEGAX v.10.1.8.^4^ Evolutionary models used were Tamura-Nei + gamma distribution (G) + invariant sites (I) for ML and Kimura two-parameter for NJ trees. Bootstrap analysis was performed with 500 replicates for the ML tree and 1,000 replicates for the NJ tree to assess branch pattern reliability (Felsenstein, 1985).

The phylogenomic tree was reconstructed by using the UBCG (v.3.0) pipeline based on 92 housekeeping genes (Na et al., 2018) using the default options. Final trees were formatted with MEGAX. It must be noted that not all *Isoptericola* species had publicly available genomes at the time the analysis was carried out.

### Biochemical, physiological, and morphological characterization

The comparative analysis of the phenotypic characteristics of 4D.3^T^ was performed in parallel with four reference strains *Isoptericola cucumis* DSM 101603^T^, *Isoptericola nanjingensis* DSM 24300^T^, *Isoptericola variabilis* DSM 10177^T^ and *Krasilnikoviella flava* DSM 21481^T^ and was carried out after 4 days of growth on the suggested DSMZ medium 92 (TSA) at 30°C unless otherwise specified. All growth assays were conducted in duplicate.

The cell morphology was examined by crystal violet stain and visualization under an optical microscope (DM2500 LED, Leica) and mycelium formation was tracked after 18 h, 48 h, and 72 h of incubation in TSA at 30°C and the same procedure for microscope visualization. At the same time, cell size was determined by measuring the minimum and maximum sizes of a random cell population (*n* = 25). The hanging-drop method was used to check the strain motility (Bernardet et al., 2002). Catalase activity was assessed by hydrogen peroxide 30% (v/v) and bubble formation was recorded as positive result. Oxidase activity was tested with the commercial Oxidase Test Stick (PanReac Applichem). Gram type test was conducted with KOH 3% (w/v) and the lack or appearance of viscosity was recorded as a Gram-negative or Gram-positive type, respectively (Halebian et al., 1981).

The ability of all the strains to grow at different temperatures (4, 12, 16, 20, 24, 30, 37, 38, 40, 42, 45°C) and salt tolerance (NaCl concentrations 0–15% at intervals of 0.5%) was assessed in TSA medium after 4 days of incubation. Growth at different pH values (4.0 to 10.0 at intervals of 1.0 pH unit) in Tryptic Soy Broth (TSB, composition in g/L: 15.0 Tryptone, 5 Soya peptone, 5 NaCl) using specific buffers at 10 mM (MES for pH 4–6, HEPES for pH 7–8, and CHES for pH 9–10) was also tested after the same incubation time (Pascual et al., 2015).

To assess the ability to grow under anaerobic and microaerophilic conditions, the BD GasPak EZ pouch system (Becton, Dickinson and Company) and the candle jar method were used, respectively.

API 20NE and API ZYM system strips (bioMérieux) along with BIOLOG GEN III MicroPlates (BIOLOG) were used according to the manufacturer’s instructions to test the assimilation of C-sources and enzymatic activities.

Analysis of cellular fatty acids for each strain was performed from a 24 h-old culture in TSB incubated at 30°C and was performed by the Spanish Type Culture Collection (CECT) by a gas chromatography system (model 6850, Agilent) and following the MIDI Microbial Identification System with the TSBA6 method (MIDI, 2008; Sasser, 1990).

### Biosurfactant synthesis assays

To perform a screening of the genus *Isoptericola* on the ability to produce biosurfactants, three complementary assays previously described in the literature (Lee et al., 2018a; Walter et al., 2010) were carried out with several species of the genus. Specifically, the CTAB assay, the emulsification index E_24_% and the hemolytic activity assay were carried out and are explained in more detail in the following paragraphs. Eight strains were tested, including the aforementioned closer strains used for the biochemical and physiological characterization *Isoptericola peretonis* sp. nov. 4D.3^T^, *Isoptericola cucumis* DSM 101603^T^, *Isoptericola nanjingensis* DSM 24300^T^, *Isoptericola* var*iabilis* DSM 10177^T^ and *Krasilnikoviella flava* DSM 21481^T^ as well as *Isoptericola chiayiensis* DSM 27643^T^*, Isoptericola halotolerans* DMS 16376^T^*, Isoptericola jiangsuensis* DMS 21863^T^ and *Isoptericola hypogeus* DSM 16849^T^ also purchased from the DSMZ (Leibniz Institut, Deutsche Sammlung von Mikroorganismen und Zellkulturen GmbH, Germany).

#### CTAB assay

This method allows the detection of anionic biosurfactants throughout the formation of a dark precipitate when the biosurfactant reacts with the cetyltrimethylammonium bromide (CTAB) (Ref. 219374, Merck KGaA, Darmstadt, Germany) (Lee et al., 2018a). The assay was conducted in duplicate. Bushnell Haas medium plates (Composition in g/L: 0.4 MgSO_4_·7H_2_O, 0.020 CaCl_2_, 1.0 KH_2_PO_4_, 1.0 K_2_HPO_4_, 1.0 NH_4_NO_3_, 0.05 FeCl_3,_ 15.0 Agar) supplemented with glucose 2% (w/v), CTAB (0.5 mg/mL) and methylene blue (0.2 mg/mL) were prepared. Precultures of all the strains were grown in 5 mL of in TSB at 30°C and agitation during 24 h, 48 h and 72 h. After each incubation time, cultures were divided in two volumes and the cell-free supernatant was obtained by centrifugation of one of the volumes (2.5 mL) of the culture at 10,000 rpm for 15 min and 1 mL of each supernatant was transferred to a new tube. A drop assay was carried out by inoculating a drop of 10 μL of each cell-free supernatant and the containing-cell culture in CTAB agar plates. As positive control, a set of dilutions of the anionic surfactant SDS of 10, 5, 1, 0.5, and 0.1% (v/v) were used as well as for the non-ionic surfactant Tween 80 10% (v/v) as negative control (non-ionic surfactant), were added in drops of 10 μL. Plates were incubated for 24 h and the production of a dark precipitate was semi-quantitatively measured by the following rule: −, no dark precipitate appeared; +, a light dark precipitate appeared; ++, a moderate precipitate appeared; +++ a strong dark precipitate appeared; ++++ a very strong dark precipitate appeared.

#### Emulsification index E_24_%

The emulsification ability of all the strains tested was measured by modifying the emulsification index E_24_% assay described by Cooper and Goldenberg (1987). The assay was conducted in duplicate. Each strain was inoculated in 30 mL TSB cultures that were incubated for 48 h at 30°C and 180 rpm. Afterwards, half of the volume was centrifuged at 10,000 rpm for 15 min to get the cell-free supernatant. The assay was set in parallel with the cell-free supernatant and the cell-containing culture, using either mineral oil or diesel as hydrocarbons. Therefore, 6 mL of the cultures or cell-free supernatants were mixed with 3 mL of the selected hydrocarbon in an assay tube. Then, they were mixed at a maximum speed by agitation in a vortex for 2 min and were allowed to stand for 24 h in vertical position at RT. The same procedure was followed for negative controls (non-inoculated TSB) and positive controls (Tween 80 2:1 (v/v) and SDS 10% (v/v) solutions), in which these solutions were used instead of the bacterial cultures. Afterwards, to calculate the E_24_% index, the height of the emulsified layer (mm) and the total height of the liquid column (mm) were measured and determined by the equation:


$$E24\%=\frac{\mathrm{Height}\;\mathrm{of}\;\mathrm{emulsified}\;\mathrm{layer}\;\left(\mathrm{mm}\right)}{\mathrm{Total}\;\mathrm{height}\;\mathrm{of}\;\mathrm{liquid}\;\mathrm{column}\;\left(\mathrm{mm}\right)}\times 100$$

#### Hemolytic activity assay

Biosurfactants have hemolytic activity. Therefore, when a biosurfactant-producing strain is grown on Blood Agar plates (BA, Ref: 0931, Condalab, Spain) a halo due to hemolysis of erythrocytes is expected (Sharma et al., 2015). The assay was conducted in duplicate. TSB cultures of 5 mL were inoculated separately with each strain and were incubated at 30°C and agitation for 48 h. After the incubation time, the Optical Density 600 nm (OD_600_) was adjusted to 1 and 10 μL drops were inoculated in BA. Also, 10 μL drops of the anionic surfactant SDS at different concentrations (10, 5, 1, 0.5, and 0.1%, v/v) as well as the non-ionic surfactant Tween 80 10% (v/v) were used as positive control. Results were measured semi-quantitatively by measuring the halo of hemolysis produced by each strain at different incubation times (24 h, 72 h, and 10 days) at 30°C following the rule: −, no hemolysis; +, incomplete hemolysis; ++, complete hemolysis with a diameter of lysis <0.5 cm; +++, complete hemolysis with a diameter of lysis >0.5 cm.

### Genome analysis for hydrocarbon degradation, rhamnolipid production and biosafety of *Isoptericola* strains

At the time of performing this work, the genome of the 12 publicly available genomes of the strains *Isoptericola peretonis* sp. nov. 4D.3^T^, *Isoptericola jiangsuensis* DMS 21863^T^*, Isoptericola dokdonensis* DS-3^T^, *Isoptericola chiayiensis* KCTC 19740^T^, *Isoptericola sediminis* JC619^T^, *Isoptericola halotolerans* KCTC 19046^T^, *Isoptericola croceus* q2^T^, *Isoptericola cucumis* CCM 8653^T^, *Isoptericola luteus* NEAU Y5^T^, *Krasilnikoviella flava* DSM 21481^T^, *Isoptericola nanjingensis* DSM 24300^T^ and *Isoptericola* var*iabilis* MX5^T^ were analyzed by bioinformatic tools for hydrocarbon degrading and rhamnolipid production activities as well as to determine some biosafety traits.

Genes related to hydrocarbon degradation were annotated with CANT-HYD (Khot et al., 2022). Only hits with an *e*-value lower than 10^−6^ were considered positive. In the case that an ORF was annotated as different genes, only the annotation with a lower *e*-value was considered. BLASTp (v. 2.0.9+) was used to determine the presence of genes *rhlA*, *rhlB* and *rhlC* which are responsible of rhamnolipid production in bacteria (*e*-value threshold = 10^−6^). The potential to produce surfactin was evaluated with both BLASTp (genes *sfrAA*, *sfrAB* and *sfrAC*) and antiSMASH (v. 7.0) (Blin et al., 2023). Genes were downloaded from UniProt along with other sequences that had at least 50% similarity to the reference sequences.

Several bioinformatic tools were applied to assess the biosafety of the strains. Specifically, ABRicate^5^ was used to find possible antimicrobial resistance (AMR) genes or virulence factors. This tool searches the proteins predicted from the genome of interest against a set of different databases: NCBI AMRFinderPlus (Feldgarden et al., 2019), CARD (Jia et al., 2017), ARG-ANNOT (Gupta et al., 2014), Resfinder (Zankari et al., 2012), MEGARES (Doster et al., 2020), EcOH (Ingle et al., 2016), PlasmidFinder (Carattoli et al., 2014), Ecoli_VF and VFDB (Chen et al., 2016). Additionally, RGI-CARD^6^ (Alcock et al., 2022) and abriTAMR^7^ were also employed to detect potential AMR genes. Finally, Bakta was used to identify biogenic amines (Schwengers et al., 2021).

## Results

### Biochemical, physiological and morphological analysis

The strain 4D.3^T^ is a Gram-positive, facultatively anaerobic and non-motile bacterium that formed primary mycelium with irregular and aggregated morphology from cocci to v-shaped rods varying in size (0.8–1.0 μm in diameter and 1–3 μm long) in the first 48 h. Homogenous cocci shape and absence of mycelium was observed after 72 h of incubation (Supplementary Figure S1). The colonies of 4D.3^T^ after 72 h, were circular and raised with 2–3 mm in diameter and appeared opaque-orange, shiny and smooth with regular edges. The strain grew well under aerobic conditions and showed less growth under microaerophilic and anaerobic conditions.

Strain 4D.3^T^ displayed a mesophilic growth, being able to grow between 12 and 42°C (optimum 30°C). It was able to grow well up to 8% of NaCl and weakly 10% (optimum 0–5%). It showed optimum growth between pH values 6–7 but it was able to grow up to pH 10. Strain 4D.3^T^ tested catalase positive and oxidase negative.

In carbon source assimilation tests using API 20 NE strips, 4D.3^T^ assimilated d-glucose, l-arabinose, d-mannose, d-mannitol, N-acetyl-glucosamine, d-maltose and potassium gluconate. In the BIOLOG GENIII assay, strain 4D.3^T^ was found to oxidize 46 out of the 71 tested carbon sources. Among them, d-sorbitol, 3-methyl glucose, d-fucose, N-acetyl-β-d-mannosamine, N-acetyl- d-galactosamine and l-serine were exclusively assimilated by 4D.3^T^ when compared with the reference strains *I. cucumis* DSM 101603^T^, *I. nanjingensis* DSM 24300^T^, *I.* var*iabilis* DSM 10177^T^ and *Krasilnikoviella flava* DSM 21481^T^ (Supplementary Table S1).

Strain 4D.3^T^ was positive in various enzymatic activities, such as alkaline phosphatase, esterases (C4 and C8), leucine arylamidase, valine arylamidase, cystine arylamidase, naphtol-AS-BI-phosphohydrolase, α-galactosidase, β-galactosidase, α-glucosidase, β-glucosidase (API ZYM), aesculin hydrolysis (API 20NE) and gelatinase. Conversely, strain 4D.3^T^ exhibited negative responses for lipase (C14), trypsin, α-chymotrypsin, acid phosphatase, β-glucuronidase, N-acetyl-β-glucosaminidase, α-mannosidase, α-fucosidase, urease, fermentation of glucose, arginine dihydrolysis and indole production. Reduction of nitrates to nitrites tested positive whereas reduction of nitrates to nitrogen tested negative. Table 1 shows differential phenotypic characters between strain 4D.3^T^ and its phylogenetically closest species.

**Table 1:** Differential phenotypic characteristics of strain 4D.3^T^ and the other closely related reference strains: 1, *Isoptericola peretonis* sp. nov. 4D.3^T^; 2, *Isoptericola cucumis* DSM 101603^T^; 3, *Isoptericola variabilis* DSM 10177^T^; 4, *Isoptericola nanjingensis* DSM 24300^T^; 5, *Krasilnikoviella flava* DSM 21481^T^.

Characteristic	1	2	3	4	5
Isolation source	Car tank lid	Cucumber root tissue	Termite hindgut	Soil	Baltic sea sediment
**Growth at/in**					
Temperature range (°C) (optimal)	12–42 (30)	12–40 (30)	12–42 (30)	12–38 (30)	12–30 (30)
NaCl tolerance (%, w/v) (optimal)	0–10 (0–5)	0–11 (0–7)	0–10 (0–5)	0–10 (0–5)	0–9 (0–5)
pH (optimal)	6–10 (6–7)	7–10 (8)	5–9 (6–9)	5–10 (6–9)	6–9 (6)
**Carbon source utilization (API 20NE)**					
Malic acid	−	+	−	−	−
**Enzymatic activity (API ZYM)**					
Valine arylamidase	w	−	w	w	w
Cystine arylamidase	w	−	w	w	w
Trypsin	−	−	−	w	−
α-galactosidase	+	−	+	w	w
N-acetyl-β-glucosaminidase	−	w	−	−	−
α-mannosidase	−	+	−	w	w

Data for all strains were obtained in the present study. +, positive; w, weak; −, negative. All strains are positive for alkaline phosphatase, esterase lipase (C4), esterase lipase (C8), leucine arylamidase, naphtol-AS-BI-phosphohydrolase, β-galactosidase, α-glucosidase, β-glucosidase, reduction of nitrates to nitrites, aesculin hydrolysis, gelatinase and the assimilation of d-glucose, l-arabinose, d-mannose, d-mannitol, N-acetyl-glucosamine, d-maltose and potassium gluconate. All strains are negative for lipase (C14), α-chymotripsin, acid phosphatase, β-glucuronidase, α-fucosidase, reduction of nitrates to nitrogen, indole production, fermentation of glucose, arginine dihydrolysis, urease and the assimilation of capric acid, adipic acid, trisodium citrate, and phenylacetic acid.

In terms of the fatty acid composition, only saturated fatty acids were detected at high concentrations as well as hydroxylated fatty acids in trace amounts. The major fatty acid of strain 4D.3^T^ was anteiso-C_15:0_ making up 59.44% of the total composition, followed by iso-C_15:0_ accounting for 29.02%. Other less abundant (<5%) fatty acids were anteiso-C_17:0_, iso-_C16:0_, C_16:0_, and iso-C_14:0_. This fatty acid pattern was very similar to that of the reference strains (Table 2) as well other members of the genus *Isoptericola* (Stackebrandt and Schumann, 2015).

**Table 2:** Cellular fatty acid profile (%) of strain 4D.3^T^ and its closest type strains.

	1	2	3	4	5
**Saturated**					
iso-C_14:0_	1.09	2.94	2.37	1.28	tr
C_14:0_	tr	1.76	1.52	1.33	1.44
iso-C_15:0_	29.02	17.75	25.14	25.56	22.73
anteiso-C_15:0_	59.44	64.44	54.17	59.17	59.88
iso-C_16:0_	2.38	3.61	7.16	4.07	3.79
C_16:0_	1.15	1.54	1.66	1.93	2.20
iso-C_17:0_	tr	tr	tr	tr	1.14
anteiso-C_17:0_	4.86	7.09	6.86	5.99	8.01
**Hydroxylated**					
C_12:0_ 2-OH	tr	tr	tr	–	–

*Strains*: 1, *Isoptericola peretonis* sp. nov. 4D.3^T^; 2, *Isoptericola cucumis* DSM 101603^T^; 3, *Isoptericola variabilis* DSM 10177^T^; 4, *Isoptericola nanjingensis* DSM 24300^T^; 5, *Krasilnikoviella flava* DSM 21481^T^. tr, trace (<1.0%); −, not detected.

### Genomic, phylogenetic and phylogenomic analysis of *Isoptericola* 4D.3^T^ sp. nov.

The 16S rRNA gene sequence of strain 4D.3^T^ obtained by Sanger sequencing was almost complete (1,356 bp, MZ562363) and 100% identity was obtained when compared with the 16S rRNA gene sequence extracted from its genome (1,527 bp), as expected. According to the EzBioCloud database, the closest type strains of 4D.3^T^ were *Isoptericola nanjingensis* H17^T^ (99.8% 16S rRNA gene sequence similarity), *Isoptericola cucumis* AP-38^T^ (99.5%) and *Isoptericola* var*iabilis* MX5^T^ (99.4%). Other type strains of the genus *Isoptericola* showed sequence similarity values of less than 99.0%.

**Figure 1:**
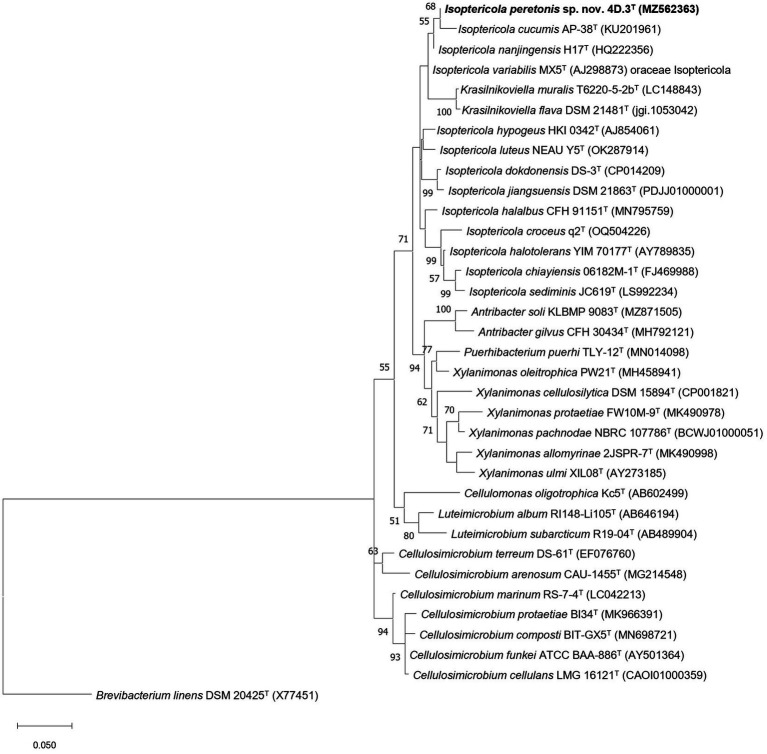
Maximum likelihood phylogenetic tree showing the taxonomic position of *I. peretonis* sp. nov. 4D.3^T^ and other related members of the families *Promicromonosporaceae* and *Cellulomonadaceae.* The evolutionary model used was Tamura-Nei + gamma distribution (G) + invariant sites (I) and bootstrap values based on 500 replicates are shown at nodes (only values >50% are shown). As outgroup, *Brevibacterium linens* DSM 20425^T^ (X77451) was used. Bar 0.050 fixed nucleotide substitutions per site.

In both the NJ and ML phylogenetic trees based on the 16S rRNA gene, strain 4D.3^T^ forms a monophyletic group with the type strain of the species *I. cucumis*, with *I. nanjingensis* and *I. variabilis* occurring as external species to this group. However, this group is not very stable considering the bootstrap values presented at the nodes (<75%). Interestingly, in both phylogenetic trees, ML and NJ, the two species of the genus *Krasilnikoviella*, *K. flava*, and *K. muralis*, are placed within the genus *Isoptericola*, appearing as a paraphyletic group. This result suggests that the members of the genus *Krasilnikoviella* should be reclassified as species of the genus *Isoptericola* (ML: Figure 1; NJ: Supplementary Figure S2).

The draft genome of the strain 4D.3^T^ has a length of 4,474,848 pb and consisted of 20 contigs, with an N50 value of 867,559 bp and a genomic DNA G + C content of 74%. The genomic G + C content of strain 4D.3^T^ was within the range described for the genus *Isoptericola*, namely 70.0–74.1% (Stackebrandt and Schumann, 2015). The predicted annotation identified 4,139 coding sequences (CDSs), out of which 2,797 were predicted as proteins with functional assignments. Additionally, 49 tRNAs and 3 rRNAs were predicted. The genome completeness was 100% and the contamination level was 0.58%, indicating that the draft genome had high-quality for further analyses (Riesco and Trujillo, 2024).

**Figure 2:**
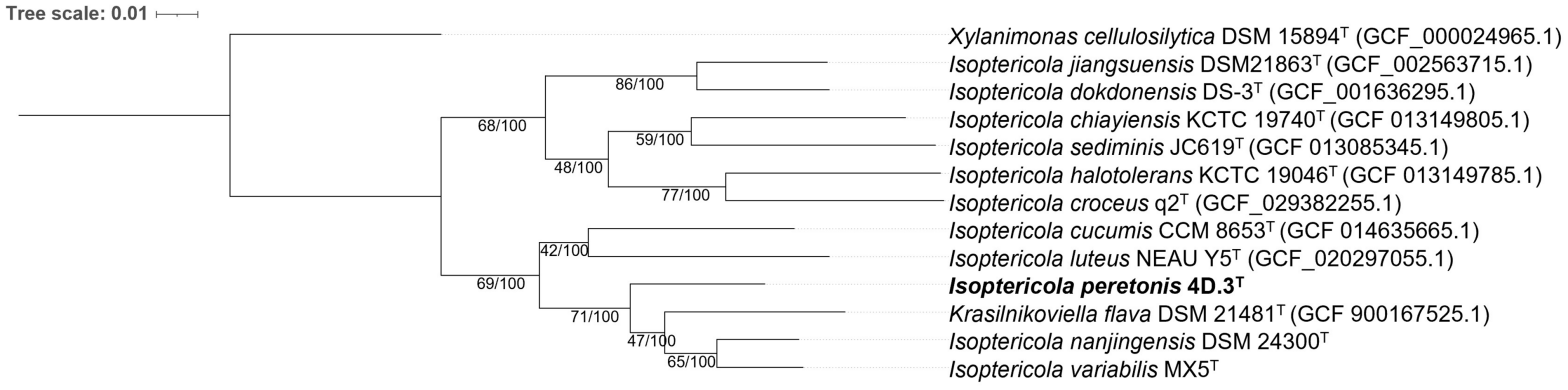
Phylogenomic tree based on a multiple alignment of 92 housekeeping gene sequences using the UBCG (v.3.0) pipeline (Na et al., 2018) showing taxonomic relationships between *I. peretonis* sp. nov. 4D.3^T^ and other related species in the family *Promicromonosporaceae.* Bootstrap analysis was performed using 100 replicates. Gene support indices (maximum value; 92 genes) and percentage bootstrap values (maximum value; 100%) are shown at branch points. Bar indicates 0.01 substitutions per nucleotide position. *Xylanimonas cellulosilytica* DSM 15894^T^ (GCF_000024965.1) was used as an outgroup.

For a more precise determination of the phylogenetic position of 4D.3^T^ within the *Isoptericola* genus, a phylogenomic tree was constructed using genomic sequences (Figure 2). Strain 4D.3^T^ formed a well-defined monophyletic group with the type strains of the species *I. nanjingensis*, *I.* var*iabilis* and *K. flava*. This clustering is stable, as proved by the high bootstrap values (100%) and the genetic support index (71 out of a total of 92 genes). The inclusion of the species *K. flava* into the genus *Isoptericola* suggests that this species should be reclassified as a member of the genus *Isoptericola*. However, as the genome of the type species of the genus *Krasilnikoviella*, namely *K. muralis*, was not publicly available, we could not confirm this hypothesis.

The genomic index ANIb between strain 4D.3^T^ and its closest relatives was calculated to be 88.22% (*K. flava*), 87.91% (*I. nanjingensis*) and 87.71% (*I. variabilis*). For the dDDH index, the values were 31.5, 30.1 and 29.8%, respectively (Table 3). As these values were lower than the thresholds for the delimitation of novel bacterial species, i.e., 95% for ANIb (Richter and Rosselló-Móra, 2009) and 70% for dDDH (Meier-Kolthoff et al., 2013), they further supported the classification of *I. peretonis* sp. nov. 4D.3^T^ as a novel species of the genus *Isoptericola* (Chun and Rainey, 2014; Riesco and Trujillo, 2024).

**Table 3:** Genome distance indexes for strain 4D.3^T^ compared to other close type strains.

	2	3	4	5	6
**ANIb**					
1. 4D.3^T^	84.44	87.71	87.91	88.22	83.86
**dDDH**					
1. 4D.3^T^	24.6 (22.2–27.0)	29.8 (27.4–32.3)	30.1 (27.7–32.6)	31.5 (29.1–34.1)	23.9 (21.6–26.4)

Average Nucleotide Identity values (ANIb) and *in silico* digital DNA–DNA Hybridization (dDDH) values together with the confidence intervals in parenthesis are shown in %. 1, *Isoptericola peretonis* sp. nov. 4D.3^T^; 2, *Isoptericola cucumis* DSM 101603^T^; 3, *Isoptericola variabilis* DSM 10177^T^; 4, *Isoptericola nanjingensis* DSM 24300^T^; 5, *Krasilnikoviella flava* DSM 21481^T^; 6, *Isoptericola luteus* NEAU Y5^T^.

### Biosurfactants production by several species from the genus *Isoptericola*

**Figure 3:**
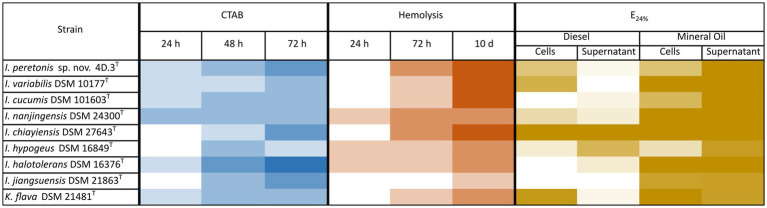
Emulsification activity by *Isoptericola peretonis* sp. nov. 4D.3^T^ and related species. Heatmap of three complementary assays to detect biosurfactants synthesis. CTAB assay: Detection of anionic surfactants by qualitative measurement of the appearance of a dark precipitate. In a blue gradient, the appearance of the dark precipitate is shown from dark blue (very strong precipitate = presence of anionic biosurfactant) to white (no precipitate = absence of anionic biosurfactant). Hemolytic assay: Semi-quantitative measurement of halo formation in Blood Agar plates due to hemolysis caused by the lysis of erythrocytes after strains growth. In a red gradient, the hemolytic activity from dark red (complete hemolysis with a lysis diameter > 0.5 cm) to white (no hemolysis). Emulsification index E_24_% assay: Quantitative measurement of the emulsification ability of a liquid culture of each strain (TSB, 48 h incubation, 30°C and 180 rpm) on diesel and mineral oil. Cell-containing culture and cell-free supernatant were tested. In a brown gradient, the E_24_% from dark brown (100% = complete emulsification) to white (0% = absence of emulsification). In all the assays positive controls displayed the maximum value and negative controls displayed the minimum value of precipitation/hemolysis/emulsification.

In order to elucidate the ability to produce biosurfactants by the majority of the species of the genus *Isoptericola*, complementary in vitro assays to check biosurfactant synthesis of strains close to the new *Isoptericola* strain 4D.3 ^T^ were performed, and results are summarized in Figure 3. Eight strains belonging to the *Isoptericola* genus as well as the *K. flava* 21481^T^ strain were included in the CTAB, Hemolytic and E_24_% assays as described in Materials and Methods section.

Overall, all the strains tested showed able to produce biosurfactants and the increase in the incubation time correlated to a higher biosurfactant production. At the same time, the CTAB and the E_24_% assays were performed with the cell-free supernatants and cell-containing cultures, to elucidate whether biosurfactants are secreted or remain embedded to the cell surface.

Regarding the CTAB assay, which reveals the production of anionic biosurfactants, such as rhamnolipids, by the formation of a dark precipitate, it showed that all strains tested were positive in anionic biosurfactants synthesis. Furthermore, supernatants tested negative for anionic biosurfactants production and only cultures containing cells presented the dark precipitate. *I. hypogeus* DSM 16849^T^ showed the most intense precipitate when the preculture was incubated 72 h at 30°C. *I. peretonis* sp. nov. 4D.3^T^, *I. varibilis* DSM 10177^T^, *I. cucumis* DSM 101603^T^ as well as *I. chiayiensis* DSM 27643^T^ displayed the highest hemolytic activity after 10 days of incubation on blood-agar plates. In this case *I. jiangjuensis* DSM 21863^T^ differed from the rest of the strains by showing a very low hemolysis ability.

In addition, the E_24_% assay, widely used to test biosurfactant production in strains of interest, was carried out using diesel and mineral oil as the organic phase that was mixed with the cell-free supernatant as well as the cell-containing culture. As shown in Figure 3, *I. chiayiensis* DSM 27643^T^ was the only one giving a complete emulsification in both, diesel and mineral oil, followed by *K. flava* DSM 21481^T^ that presented a 100% emulsification index in the cell-containing cultures. On average, strains presented a higher E_24_% in diesel when cells were present, except for *I. hypogeus* DSM 16849^T^ which supernatant had more emulsification capacity. Regarding the new species *I. peretonis* sp. nov. 4D.3^T^, both the supernatant and the cell-containing culture showed a moderate-high E_24_% index.

### In silico analysis of hydrocarbon degradation and biosurfactant production by *Isoptericola*

**Figure 4:**
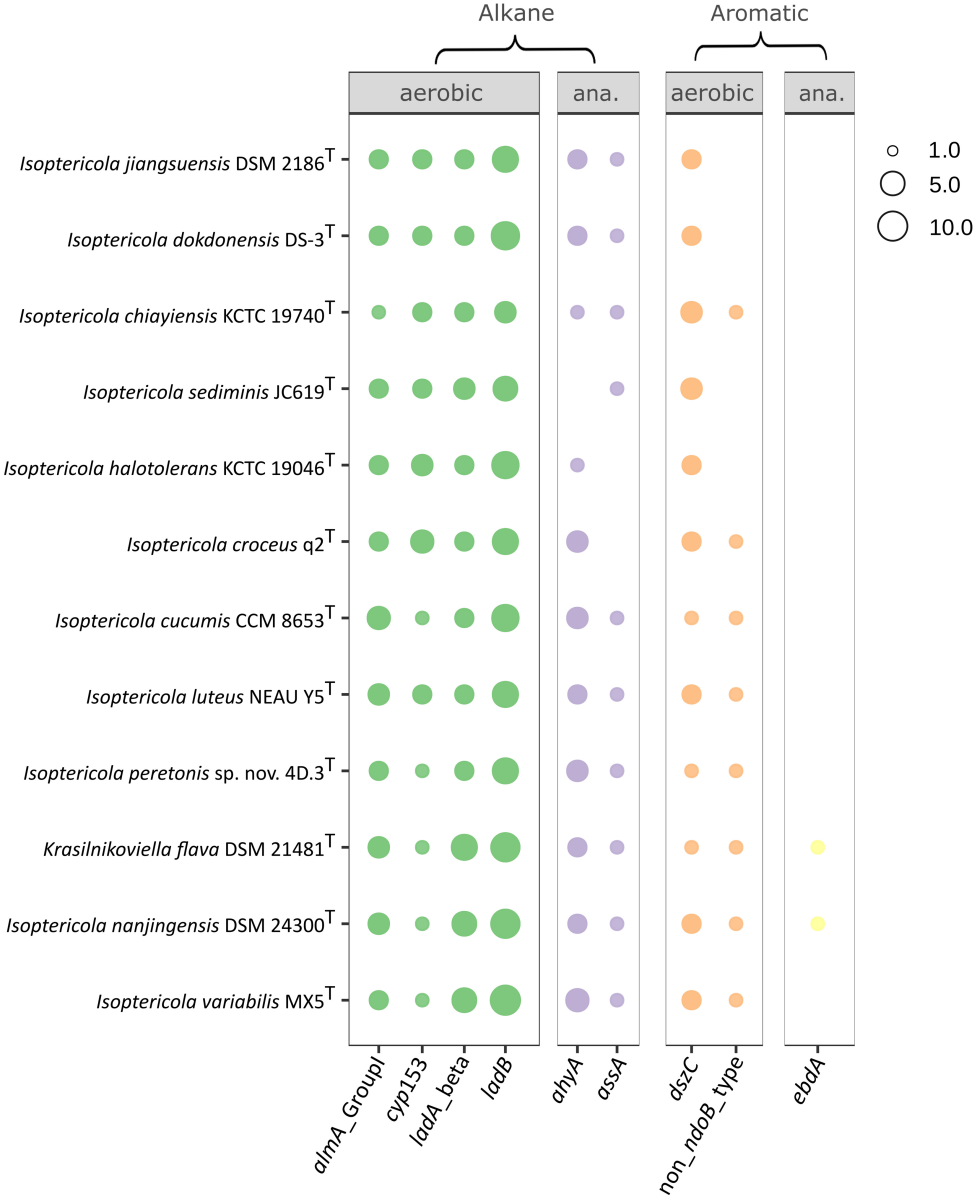
Hydrocarbon degradation capabilities of *Isoptericola* species, including *K. flava*, as deduced from the CANT-HYD annotation of each genome. Only genes with at least one hit in one genome are shown (*e*-value threshold < 10^−6^). The size of the point is proportional to the number of gene copies present in the genome. In cases where an ORF was annotated as multiple genes, only the hit with the lowest *e*-value was considered. Panels show genes implicated in aromatic and alkane hydrocarbon degradation in aerobic or anaerobic conditions. Genomes in the y axis follow the order as they appear in the phylogenomic tree (Figure 2). “ana.”: anaerobic.

All the publicly available genomes of *Isoptericola* species, including *K. flava*, were analyzed to elucidate the ability of these microorganisms to degrade aromatic hydrocarbons and alkanes (Figure 4). All the studied species contained four genes involved in the aerobic degradation of alkanes: *almA* (Group I), *cyp153*, *ladA* (beta subunit), and *ladB*. *almA* (Group I) and *cyp153* encode the enzymes flavin-binding monooxygenase and cytochrome P450 alkane hydroxylase, respectively, which are involved in the degradation of C20-C32 (*almA*) and C5-C13 (*cyp153*) alkanes. The degradation of C15-C36 alkanes would be mediated by the *ladA* (beta) and *ladB* genes, which encode long-chain alkane monooxygenases and were found in multiple copies in the genomes. However, the similarity between copies was low. For example, in the case of ORFs assigned as *ladA* or *ladB* in the *I. peretonis* genome, the similarity between ORFs annotated as the same gene was less than <40% in all cases, according to BLASTp.

All the species, except *I. croceus* and *I. halotolerans*, presented a 1-methylalkyl (alkyl) succinate synthase (gene *assA*). In contrast, *ahyA*, a gene that encodes a putative alkane C2 methylene hydroxylase, was present in all the species except for *I. sediminis*. Both enzymes are involved in the anaerobic degradation of alkanes.

Regarding the aerobic degradation of aromatic compounds, all the genomes contained copies of *dszC,* which encodes the dibenzothiophene desulfurization enzyme C. *I. jiangsuensis, I. dokdonensis, I. sediminis,* and *I. halotolerans* were the only species lacking non-NdoB type naphthalene dioxygenase alpha. Finally, *K. flava* and *I. nanjingensis* were the only species with the potential ability to produce an ethylbenzene dehydrogenase, which catalyzes the anaerobic oxidation of ethylbenzene to (S)-1-phenylethanol (Rabus et al., 2005).

The organization of genes involved in hydrocarbon degradation revealed no evidence of complex gene clusters. This was anticipated, as many pathways for hydrocarbon degradation remain incomplete, with accessory functions still unidentified. Moreover, most genomes analyzed were draft assemblies rather than fully closed genomes, limiting the detection of such clusters. Therefore, further research is necessary to clarify this aspect. Nevertheless, simpler gene organization patterns were observed. For example, the *almA* and *cyp153* genes appeared consecutively in all *Isoptericola* genomes, suggesting a linked inheritance, evolution and regulation of their functions in degrading C20-C32 (*almA*) and C5-C13 (*cyp153*) alkanes. A similar arrangement was identified for the *ahyA* gene. Several hits consistent with this gene were found adjacent to each other in different *Isoptericola* genomes, often separated by one or more genes. These hits likely represent distinct subunits of the same enzyme, as they were annotated by Prokka as “Nitrate reductase alpha subunit” and “Assimilatory nitrate reductase catalytic subunit.” In contrast, the *ladA* (beta subunit) and *ladB* genes exhibited a more complex organization. For instance, in *I. peretonis* sp. nov. 4D.3^T^, seven of the eight identified copies of these genes were scattered across different contigs. However, an interesting pattern was observed in *I.* var*iabilis*, *I. nanjingensis*, and *K. flava*. In these species, a genomic region (20–30 genes in size) contained two adjacent copies of *ladA* (beta subunit), two adjacent copies of *ladB*, and a single copy of *ladA* (beta subunit), suggesting a conserved gene organization in this group.

Regarding rhamnolipids biosynthesis, a well-known family of biosurfactants, starts with the conversion of β-hydroxydecanoyl-ACP to 3-(3-hydroxyalkanoyloxy) alkanoic acid (HAA), which is mediated by RhlA (Chong and Li, 2017). BLASTp revealed that *rhlA* homologs were present in *I. chiayiensis*, *I. croceus*, *I. cucumis*, *I. halotolerans*, *I. sediminis*, *K. flava*, *I. peretonis* sp. nov. 4D.3^T^ and *I. nanjingensis* genomes, but not in *I. variabilis* and *I. jiangsuensis*, which also showed surfactant activity (Figure 3). Genes homologous to those responsible for rhamnosyltransferase synthesis (*rhlB* and *rhlC*) were also found in several *Isoptericola* genomes (Supplementary Dataset S1). These genes catalyze both mono- and di-rhamnolipid synthesis (Pardhi et al., 2022).

The complete gene cluster responsible for surfactin synthesis (*sfrAA*, *sfrAB*, and *sfrAC*) (Pardhi et al., 2022) was not found in any genome. However, antiSMASH results showed that all the *Isoptericola* species, except for *I. sediminis*, *I. jangsuensis, I. dokdonensis* and *I. chiayensis,* were putatively able to synthesize alkylresorcinol, a phenolic lipid with amphiphilic characteristics (Baerson et al., 2010).

Regarding the strains biosafety analysis, genes responsible for the vancomycin resistance were detected in all the species in the phylogenetic cluster comprising *I. peretonis* sp. nov. 4D.3^T^, *I.* var*iabilis*, *I. nanjingensis*, *K. flava*, *I. luteus* and *I. cucumis*, although the average percentage of identity to the closest database hit was ~85% (Supplementary Dataset S2). These genes were absent in other *Isoptericola* species. Finally, genes coding for chloramphenicol resistance were also present in *I. peretonis* sp. nov. 4D.3^T^, *I. variabilis*, *I. nanjingensis* and *K. flava*, but the similarity to the closest database hit was relatively low (~79%).

## Discussion

The genus *Isoptericola* has expanded considerably since its first description when *Cellulosimicrobium variable* was reclassified as *I. variabilis* 20 years ago (Stackebrandt et al., 2004). With the present study, we add to the genus a new *Isoptericola* species, *Isoptericola peretonis* sp. nov. 4D.3^T^, which was isolated from a car tank lid, an urban environment rich in hydrocarbons and showed the ability to degrade up to 37% of diesel after 1-week incubation in minimal medium (Vidal-Verdú et al., 2022). Thus, to the best of our knowledge, this is the first description of a new type species in the genus that has been isolated from oil-polluted environments and characterized in detail as a diesel-degrading bacterium.

The phylum *Actinomycetota* (formerly *Actinobacteria*), to which *Isoptericola* belongs, is widely known for its ability to produce secondary metabolites such as antibiotics and extracellular—usually hydrolytic—enzymes, as well as for its ubiquity in a wide range of ecological environments (Barka et al., 2016). In particular, the actinomycetes class has been one of the main sources of biotechnologically relevant activities in the 21st century, not only because of their antimicrobial repertoire, but also because of other industrial applications that are increasingly gaining interest (de Souza Rodrigues et al., 2024; Izhar et al., 2024).

In this context, *Isoptericola* species have been described as a source of interesting activities that may play a role in biotechnological applications. On the one hand, *I.* var*iabilis*, the type species of the genus, is the most studied species within the taxon. It has been described as an efficient cellulolytic bacterium (Azizi et al., 2015; Ventorino et al., 2015), and amylolytic activity was also described for the first time in this species by Pascon et al. (2011). Interestingly, Krishnan et al. (2021) discovered the ability of *I. variabilis* to produce PHB, a natural polymer with potential to replace synthetic polymers in a wide range of applications. In addition, a gene coding for a glyphosate-resistant version of the enzyme 5-enolpyruvylshikimate-3-phosphate synthase was discovered in its genome which proved to confer high glyphosate tolerance in transgenic rice (Cui et al., 2016; Wen et al., 2021). On top of that, the *I.* var*iabilis* RGT01 strain was shown to harbor a wide range of nitrile-hydrolyzing activities with potential in the bioremediation of nitriles, a widespread industrial residue that pollutes natural environments (Kaur G. et al., 2014).

On the other hand, to a lesser extent, other species of the genus have also shown interesting activities. For example, the alginate lyase activity has recently gained interest to obtain unsaturated alginate, which has been shown to be active in biological functions such as stimulation of plant defense responses and plant growth, suppression of IgE for anti-allergy properties, and even anti-tumor and antioxidant properties among others (Chen et al., 2018). In this sense, some studies have described the species *I. halotolerans* as a good source of these alginate lyases (Chen et al., 2018; Dou et al., 2013; Wang et al., 2017; Zhu et al., 2018). Chitinase activity has also been found in *I. jiangsuensis*, which harbors the Is-ChiA and Is-ChiB chitinases (Wu et al., 2011). *I. dokdonensis* and *I. salitolerans* have also been described as source of efficient cellulases and amylases, respectively (Bae et al., 2021). Furthermore, Su et al. (2021) described for the first time in this genus, the production of new flavonoids, terpenoids and other bioactive metabolites in *I. chiayiensis* not previously isolated from other natural sources. Finally, some isolates have been found to promote plant growth due to their phosphate-solubilizing ability (Dastager and Damare, 2013), and their antimicrobial potential has also been suggested for some *Isoptericola* sp. isolates (Bibi et al., 2017; Dong et al., 2017; Girão et al., 2019; Kaushik et al., 2021).

It is not only the potential for activities of interest to the industry and its metabolic diversity that is important, but it should also be noted that this is a robust genus that can tolerate a wide range of growth conditions, making it even more interesting for biotechnological applications. Our findings align with previous evidence indicating the genus’ adaptability to varying oxygen levels and temperature, salinity, and pH ranges (Table 1) (Schumann and Stackebrandt, 2014). Of particular interest is the new strain *I. peretonis* sp. nov 4D.3^T^, which exhibits among the widest ranges of tolerance to growth conditions within other strains of the genus. It can grow in a range from 12 to 42°C, which is considered mesophilic. It can also tolerate salt concentrations from 0 up to 10% NaCl, which is classified as halotolerant. Additionally, it can grow in a pH range of 6 to 10, which is considered neutrophilic since its optimal growth occurs in a pH range of 6 to 7. However, it can also tolerate alkaline conditions. This new strain is also able to grow in aerobic, microaerophilic and anaerobic conditions and was able to oxidize the widest amount of carbon sources in the BIOLOG GENIII assay (46 out of the 71 carbon sources tested, Supplementary Table S1), when compared with the reference strains *I. cucumis* DSM 101603^T^, *I. nanjingensis* DSM 24300^T^, *I. variabilis* DSM 10177^T^ and *K. flava* DSM 21481^T^ in this study.

Furthermore, the taxonomic evidence provided in this work leads to the conclusion that further analysis must be conducted to validate the reclassification of the genus *Krasilnikoviella*, currently consisting of two species, *K. muralis* and *K. flava*,^8^ into the *Isoptericola* genus.

In silico analysis revealed the hydrocarbon-degrading potential of the genus *Isoptericola*. All the species contained multiple copies of putative genes involved in the degradation of a broad range of long-chain alkanes (from C5 to C36), which are the main components of crude oil (Feng et al., 2007). Genes *ladA* and *ladB* are commonly found in *Geobacillus* species, although *ladA* has been also described in a member of *Actinomycetes*, *Amycolicicoccus subflavus* (Nie et al., 2013). These genes encode alkane monooxygenases that convert long-chain alkanes to corresponding primary alcohols (Boonmak et al., 2014; Feng et al., 2007; Tourova et al., 2016), and they are found in multiple copies in the *Isoptericola* genomes, as also reported for *Geobacillus* (Boonmak et al., 2014). The relatively low similarity between paralogous genes annotated as *ladA* and *ladB* suggests that *Isoptericola* genomes contain a wide set of enzymes that may differ in substrate preference or other kinetic properties. According to CANT-HYD analysis, flavin-binding monooxygenase (*almA*) is also present in the members of the *Isoptericola* genus, providing a larger repertoire of enzymes capable of degrading long-chain hydrocarbons (Throne-Holst et al., 2007). Moreover, all the species analyzed may be able to use shorter alkanes thanks to *cyp153*. This gene has been found in several bacterial species, such as *Alcanivorax* (Nodate et al., 2006), *Acinetobacter* (Maier et al., 2001) or *Mycobacterium* (Van Beilen et al., 2006). It encodes the cytochrome P450 alkane hydroxylase, which is highly specific for medium-chain length alkanes (C6-C14) (Liang et al., 2016). The presence of a combination of some of these genes has been reported in previous studies. For example, *Alcanivorax dieselolei* strain B-5 and *A. hongdengensis* A-11-3 contain a combination of *almA* and a homolog of *cyp153*, in addition to several copies of *alkB* (absent in *Isoptericola*) (Liu et al., 2011; Wang and Shao, 2012). On the other hand, *Amycolicicoccus subflavus* DQS3-9A1^T^ contains *alkB*, *cyp153* and *ladA* (Nie et al., 2013). Most *Isoptericola* species, including *I. peretonis* sp. nov. 4D.3^T^, also showed the potential to degrade alkanes under anaerobic conditions thanks to genes *ahyA* and *assA*. The latter catalyzes the addition of alkanes to fumarate, forming alkyl-substituted succinates that can be further metabolized (Callaghan et al., 2008). Finally, the genus *Isoptericola* could degrade dibenzothiophene thanks to dibenzothiophene desulfurization enzyme C (Piddington et al., 1995), and many of its species could use naphthalene due to the presence of non-*ndoB* type naphthalene dioxygenase alpha (Khot et al., 2022). Although the present study focuses on in silico predictions of hydrocarbon degradation by *Isoptericola* strains, we showed in a previous work that the new strain *I. peretonis* 4D.3^T^ was able to degrade up to 37% of diesel after 1 week of incubation (Vidal-Verdú et al., 2022). This diesel-degradation efficiency was lower than other well-known degraders such as *Pseudomonas reidholzensis* (61%), *P. lutea* (47%), *Achromobacter deleyi* (47%) and *Bacillus megaterium* (42%) under the same conditions in the study. Hence, further quantification on the hydrocarbon degradation ability of *Isoptericola* strains is needed to confirm their potential.

On the other hand, the most studied families of biosurfactants are surfactins and rhamnolipids (Pardhi et al., 2022), both classified as anionic biosurfactants (Sass et al., 2023). As described in previous literature, microbial biosurfactants are either secreted or can remain associated to the cell surface (Sharma et al., 2022), fact that is in accordance with the in vitro E_24_% assay, in which both the cell-containing cultures and cell-free supernatants showed emulsification capacity as well as in the CTAB assay, in which only the cell-containing cultures showed precipitate formation. According to our genomic analysis, some *Isoptericola* species have the potential to synthesize the latter, which is in concordance with the fact that all the *Isoptericola* species tested in vitro in the CTAB assay showed the ability to produce anionic biosurfactants. Genes related to surfactin synthesis were not detected in any of the *Isoptericola* genomes analyzed. Rhamnolipids are usually produced by *Pseudomonas* and *Burkholderia* (Chong and Li, 2017), although their biosynthesis has also been reported in *Actinomycetes* strains such as *Streptomyces* sp. ISP2-49E (Yan et al., 2014), *Renibacterium salmoninarum* 27BN (Christova et al., 2004) and *Nocardioides* sp. A-8 (Vasileva-Tonkova and Gesheva, 2005). In fact, Lee et al. (2018a) described the production of the dirhamnolipid Rha-Rha-C6-C6:1, and six minor rhamnolipids by *I. chiayiensis* 103-Na4. *I. chiayiensis* DSM 27643^T^ showed a remarkable biosurfactant activity in CTAB, hemolysis and E_24_% assays, and homologs to both *rhlA* and *rhlC* were found in its genome. However, the rhamnosyltransferase responsible for the synthesis of mono-rhamnolipids (*rhlB*) could not be detected in this species. In addition, *rhlA* was not identified in *I.* var*iabilis* and *I. jiangsuensis*, which also exhibited surfactant properties in vitro. This could mean that either the mechanism of rhamnolipid production is different in some *Isoptericola* species, or that the surfactant effect observed is due to the complementary action of other metabolites not identified in this analysis. For instance, the genes responsible for the production of the amphiphilic compound alkylresorcinol were detected in several *Isoptericola* members, including *I. peretonis* sp. nov. 4D.3^T^.

Overall, these results highlight the metabolic diversity of *Isoptericola* in terms of hydrocarbon degradation and biosurfactant production, which explains the adaptation of the species *I. peretonis* sp. nov. 4D.3^T^ to oil-polluted environments. Nevertheless, it must be noted that all the functions have been predicted in silico and only a preliminary assay has been carried out regarding biosurfactant synthesis, so future experimental work is needed to check if all these enzymes are actually active and to confirm their functionality in either biosurfactant synthesis and aerobic or anaerobic hydrocarbon degradation. Therefore, due to the metabolic, biochemical and physiological robustness of the genus *Isoptericola* we propose it as an interesting chassis for synthetic biology and industrial applications within *Actinomycetota*. In particular, we highlight the new strain *I. peretonis* sp. nov. 4D.3^T^, proved as able to degrade hydrocarbons and produce biosurfactants, as an interesting candidate for bioremediation strategies.

### Description of *Isoptericola peretonis* sp. nov. 4D.3^T^

*Isoptericola peretonis* (pe.re.to’nis. N.L. gen. n. *peretonis*, named in honor of Juli Peretó, Professor of Biochemistry and Molecular Biology at the University of Valencia, researcher and teacher of metabolism and evolution for generations of students).

Cells are Gram-positive and present an irregular and aggregated morphology from cocci to v-shaped rods (0.8–1.0 μm in diameter and 1–3 μm long) and non-motile. Colonies are circular and raised, 2–3 mm in diameter and appeared opaque orange, shiny and smooth with regular edges. Growth occurs at 12–42°C (optimum at 30°C) and pH 6–10 (optimum 7), and it does not require NaCl for growth (tolerates up to 10%). The type strain is facultatively anaerobic, growing optimally under aerobic conditions but is also capable of growing under microaerophilic and anaerobic conditions to a lesser extent. Positive for catalase and negative for oxidase. Alkaline phosphatase, esterase (C4), esterase lipase (C8), leucine arylamidase, valine arylamidase, cystine arylamidase, naphthol-AS-BI-phosphohydrolase, α-galactosidase, β-galactosidase, α-glucosidase, β-glucosidase, aesculin hydrolysis and gelatinase activities are detected. Lipase (C14), trypsin, α-chymotrypsin, acid phosphatase, β-glucuronidase, N-acetyl-β-glucosaminidase, α-mannosidase, α-fucosidase, urease, fermentation of glucose, arginine dihydrolysis and indole production are not detected. Nitrate is reduced to nitrite. According to API 20 NE test kits, this species assimilates d-glucose, l-arabinose, d-mannose, d-mannitol, N-acetyl-glucosamine, d-maltose and potassium gluconate and does not assimilate capric acid, adipic acid, malic acid, trisodium citrate and phenylacetic acid. On BIOLOG GENIII MicroPlates, this species is positive for the utilization of d-raffinose, α-d-glucose, d-sorbitol, gelatin, pectin, *p*-hydroxyphenylacetic acid, Tween 40, dextrin, α-d-lactose, d-mannose, d-mannitol, glycyl-l-proline, d-galacturonic acid, d-maltose, d-melibiose, d-fructose, d-arabitol, l-alanine, d-trehalose, β-methyl-d-glucoside, d-galactose, d-gluconic acid, l-lactic acid, β-hydroxy-d,l-butyric acid, d-cellobiose, d-salicin, 3-methyl-d-glucose, glycerol, gentiobiose, *N*-acetyl-d-glucosamine, d-fucose, d-glucose-6 phosphate, l-glutamic acid, acetoacetic acid, sucrose, *N*-acetyl-β-d-mannosamine, l-fucose, d-fructose-6-phosphate, l-histidine, d-turanose, *N*-acetyl-d-galactosamine, l-rhamnose, acetic acid, stachyose, inosine and l-serine. On BIOLOG GENIII MicroPlates, this species is negative for assimilation of methyl pyruvate, *γ*-aminobutyric acid, l-galactonic acid lactone, d-lactic acid methyl ester, α-hydroxybutyric acid, *myo*-inositol, l-arginine, l-aspartic acid, d-glucuronic acid, citric acid, α-keto-butyric acid, glucuronamide, α-keto-glutaric acid, mucic acid, d-malic acid, propionic acid, d-aspartic acid, l-pyroglutamic acid, quinic acid, l-malic acid, *N*-acetyl-neuraminic acid, d-serine, d-saccharic acid, bromo-succinic acid and formic acid. The major cellular fatty acids are anteiso-C_15:0_ and iso-C_15:0_, followed by anteiso-C_17:0_, iso-C_16:0_, C_16:0_ and iso-C_14:0_.

The type strain is designated 4D.3^T^ (=CECT 30740^T^ = DSM 115117^T^) and was isolated from a car tank lid in Valencia (Spain). The genomic DNA has a G + C content of 74.0%. The DDBJ/ENA/GenBank accession number for the 16S rRNA gene sequence is MZ562363 and the GenBank genome accession number is JALQCY000000000.

## Data Availability

The datasets presented in this study can be found in online repositories. The names of the repository/repositories and accession number(s) can be found in the article/Supplementary material.

## Footnotes

^1^ https://lpsn.dsmz.de/genus/isoptericola

^2^ https://www.ezbiocloud.net/

^3^ https://www.patricbrc.org

^4^ http://www.megasoftware.net/

^5^ https://github.com/tseemann/abricate

^6^ https://github.com/arpcard/rgi

^7^ https://github.com/MDU-PHL/abritamr

^8^ https://lpsn.dsmz.de/genus/krasilnikoviella
