# Supplementary material for: Assessing hydrocarbon degradation capacity of Isoptericola peretonis sp. nov. and related species: a comparative study
Source: Front Microbiol. 2025 Feb 5;16:1471121. doi: 10.3389/fmicb.2025.1471121 (PMC11839211; doi:10.3389/fmicb.2025.1471121)
Supplement: Supplementary file 1 [file Supplementary_file_1.docx]

Supplementary Material

Assessing hydrocarbon degradation capacity of *Isoptericola* *peretonis* sp. nov. and related species: a comparative study

Àngela Vidal-Verdú^1*^, Adriel Latorre-Pérez^2^, Javier Pascual^2^, Ruth Mañes-Collado^1^, Aitana Nevot-Terraes^1^, Manuel Porcar^1,2*^


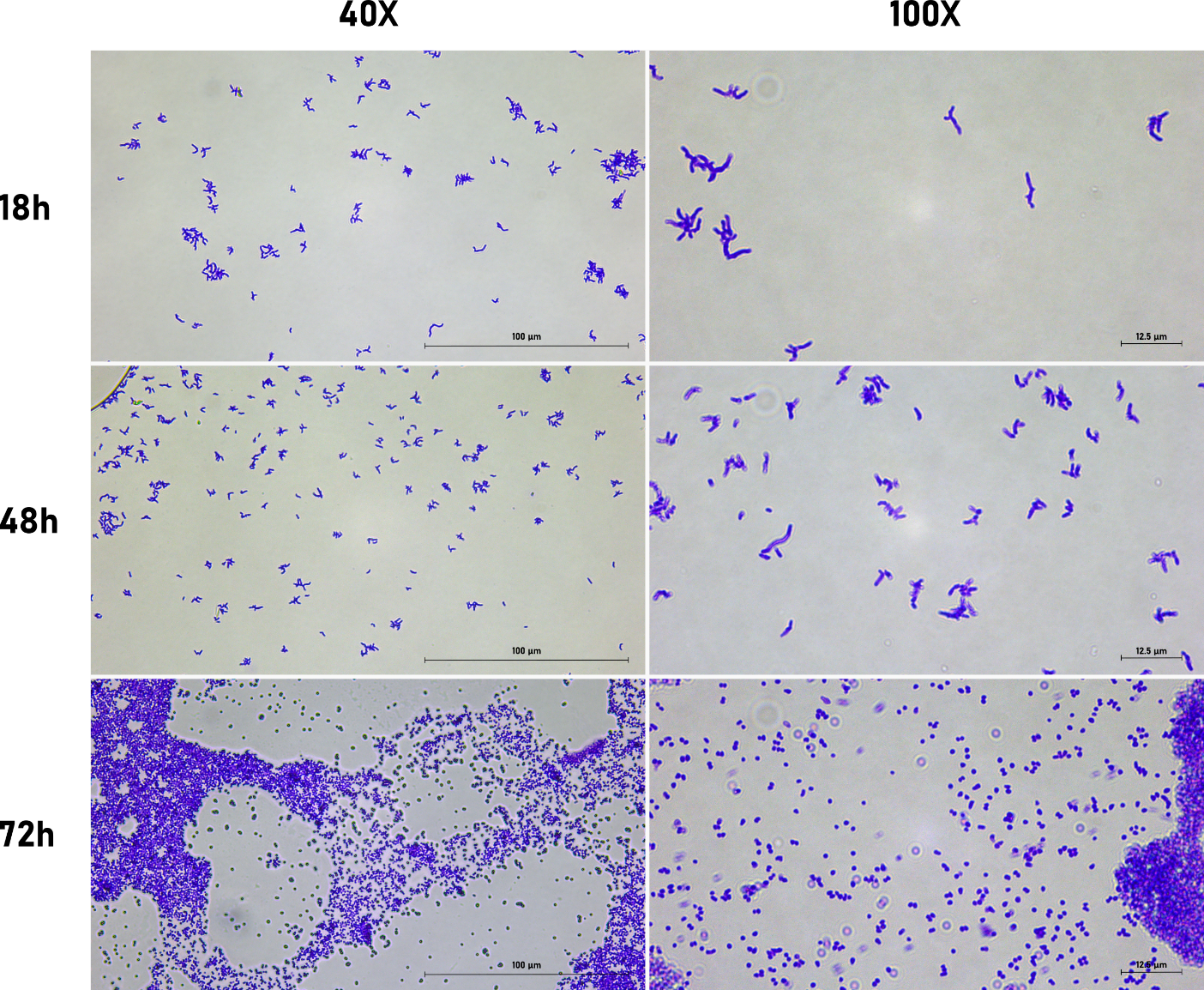


## Supplementary Figure S1. Primary mycelium formation in *Isoptericola peretonis* sp. nov. 4D.3^T^ in the first 48 h of incubation in TSA at 30ºC. At 72 h of incubation homogeneous cocci shape and absence of mycelium was observed. Images were taken in an optical microscope by using the 40X (left) and 100X (right) objectives. Cells stained with crystal violet.

**
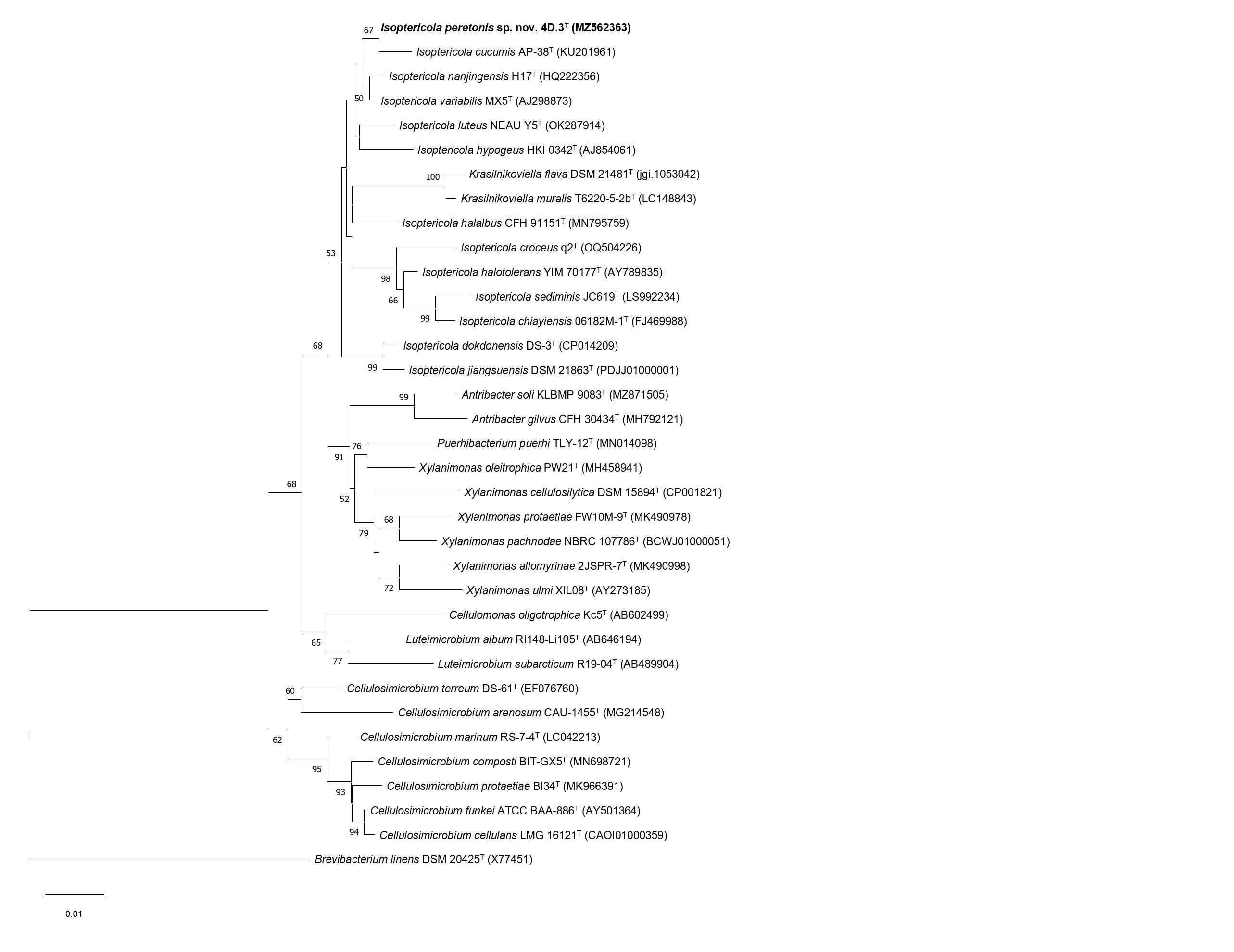
**

**Supplementary Figure S2. Neighbor-joining phylogenetic tree based on 16S rRNA gene sequences showing the position of *I. peretonis* 4D.3^T^.** The Kimura two parameter (K2P) evolutionary model of nucleotide substitution was applied. Bootstrap values (%) based on 1000 replicates are indicated at branch points (values under 50% are not shown). *Brevibacterium linens* DSM 20425^T^ (X77451) was used as an outgroup. Bar 0.01 fixed nucleotide substitutions per site.

**Supplementary Table S1.** Carbon source utilization analysis using Gen III Micro Plates of *Isoptericola peretonis* 4D.3^T^ and the other closely related reference strains: *Isoptericola cucumis* DSM 101603^T^; *Isoptericola variabilis* DSM 10177^T^; *Isoptericola nanjingensis* DSM 24300^T^; *Krasilnikoviella flava* DSM 21481^T^. Data for all strains were obtained in the present study. +, positive; -, negative. All strains are positive for D-raffinose, *α*-D-glucose, gelatin, pectin, p-hydroxy-phenylacetic acid, Tween 40, dextrin, D-mannose, D-mannitol, D-maltose, D-melibiose, D-fructose, D-arabitol, D-trehalose, D-galactose, D-gluconic acid, L-lactic acid, D-cellobiose, glycerol, gentiobiose, N-acetyl-D-glucosamine, acetoacetic acid, sucrose, D-turanose, L-rhamnose, acetic acid and stachyose. All strains are negative for *γ*-amino-butyric acid, L-galactonic acid lactone, *α*-hydroxy-butyric acid, myo-inositol, L-arginine, L-aspartic acid, citric acid, *α*-keto-butyric acid, glucuronamide, mucic acid, D-malic acid, D-aspartic acid, L-pyroglutamic acid, quinic acid, D-serine, D-saccharic acid and formic acid.

| **Characteristic** | ***Isoptericola* *peretonis* 4D.3^T^** | ***Isoptericola cucumis* DSM 101603^T^** | ***Isoptericola variabilis* DSM 10177^T^** | ***Isoptericola nanjingensis* DSM 24300^T^** | ***Krasilnikoviella flava* DSM 21481^T^** |
| --- | --- | --- | --- | --- | --- |
| D-Sorbitol | + | - | - | - | - |
| *α*-D-Lactose | + | - | + | + | + |
| Glycyl-L-proline | + | + | - | + | + |
| D-Galacturonic acid | + | + | - | - | + |
| Methyl pyruvate | - | - | - | - | + |
| L-Alanine | + | + | - | - | + |
| D-Lactic acid methyl ester | - | + | - | - | + |
| *β*-Methyl-D-glucoside | + | - | + | + | + |
| *β*-Hydroxy-D,L-butyric acid | + | + | - | + | + |
| D-Salicin | + | - | + | + | + |
| 3-Methyl-D-glucose | + | - | - | - | - |
| D-Glucuronic acid | - | + | - | - | + |
| D-Fucose | + | - | - | - | - |
| D-Glucose-6PO_4_ | + | + | - | - | + |
| L-Glutamic acid | + | + | + | - | + |
| *α*-Keto-glutaric acid | - | - | - | - | + |
| N-Acetyl-*β*-D-mannosamine | + | - | - | - | - |
| L-Fucose | + | + | + | - | + |
| D-Fructose-6PO_4_ | + | + | - | - | - |
| L-Histidine | + | - | + | - | + |
| Propionic acid | - | + | - | - | - |
| N-Acetyl-D-galactosamine | + | - | - | - | - |
| L-Malic acid | - | + | - | - | - |
| N-Acetyl neuraminic acid | - | + | - | - | - |
| Inosine | + | + | + | - | + |
| L-serine | + | - | - | - | - |
| Bromo-succinic acid | - | + | - | - | - |
